# Recent Options and Techniques to Assess Improved Bioavailability: In Vitro and Ex Vivo Methods

**DOI:** 10.3390/pharmaceutics15041146

**Published:** 2023-04-04

**Authors:** Liza Józsa, Dániel Nemes, Ágota Pető, Dóra Kósa, Réka Révész, Ildikó Bácskay, Ádám Haimhoffer, Gábor Vasvári

**Affiliations:** 1 Department of Pharmaceutical Technology, Faculty of Pharmacy, University of Debrecen, Nagyerdei St. 98, H-4032 Debrecen, Hungary; 2 Institute of Healthcare Industry, University of Debrecen, Nagyerdei St. 98, H-4032 Debrecen, Hungary

**Keywords:** in vitro methods, cell culture, ex vivo methods, bioavailability

## Abstract

Bioavailability assessment in the development phase of a drug product is vital to reveal the disadvantageous properties of the substance and the possible technological interventions. However, in vivo pharmacokinetic studies provide strong evidence for drug approval applications. Human and animal studies must be designed on the basis of preliminary biorelevant experiments in vitro and ex vivo. In this article, the authors have reviewed the recent methods and techniques from the last decade that are in use for assessing the bioavailability of drug molecules and the effects of technological modifications and drug delivery systems. Four main administration routes were selected: oral, transdermal, ocular, and nasal or inhalation. Three levels of methodologies were screened for each category: in vitro techniques with artificial membranes; cell culture, including monocultures and co-cultures; and finally, experiments where tissue or organ samples were used. Reproducibility, predictability, and level of acceptance by the regulatory organizations are summarized for the readers.

## 1. Introduction

Over the past decade, efforts have been made to develop reliable in vitro and ex vivo models that mimic all relevant biological barriers in the preclinical drug testing [1]. This phenomenon was stimulated by the need to rationalize drug development and research processes and make the results more reproducible [2]. In addition, significant scientific efforts have been made to discover alternative methods of drug development and testing for ethical reasons, as animal welfare has become a major concern, not only in society but also in the scientific field [3]. In 1959, Russell and Burch defined the “3R” rule (Replace, Reduce, Refine), which sets out the principles for the more ethical use of animals in product testing and scientific research [4]. Animal experiments are often carried out to determine the pharmacokinetics and toxicological data of drugs before the clinical trials; however, the regulatory authorities (e.g., EMA, FDA) enforce the replacement of animal testing and suggest the use of in vitro or ex vivo models because of ethical reasons. The use of these non-animal methods makes it possible to reduce the number of animals involved in animal experiments, refine the methods, and even replace the animals, thereby contributing to the implementation of the 3R principles and giving the potential to further minimize animal testing in preclinical research [2]. Furthermore, many animal tests are simply too costly, take too long, and provide misleading results.

Many techniques and models are successfully used at different stages of drug discovery and development, including in silico, in vitro, ex vivo, and in vivo methods [5]. The present review provides an overview of the characterization and application of novel in vitro and ex vivo methods and cell cultures used in the development and evaluation of new oral, dermal, nasal, and ocular formulations. The development of technology provided the opportunity for in vitro and ex vivo methods to become increasingly widespread. The correlation between the human data and the preclinical data obtained from these models is critical for drug design and development. The accuracy of predicting clinical outcomes is largely determined by the extent to which these models mimic the given part of the human body. Therefore, significant efforts were made to create an environment as close to humans as possible [2,6].

The use of the novel in vitro methods described in this review may lead to better in vitro-in vivo extrapolation (IVIVE) outcomes. Several models are available to screen and predict oral, transdermal, nasal, and even ocular bioavailability of an active pharmaceutical ingredient (API) at different stages of drug discovery and development. Among the latest in vitro assays for the investigation of drug permeability are the parallel artificial membrane permeability assays (PAMPA), which can be used to study both oral and transdermal (skin-PAMPA) dosage forms [7]. PAMPA assays are cost-effective, reliable, robust, quick, reproducible, and high-throughput experiments that predict the passive transcellular permeability of the APIs. According to the type of investigation, the biomimetic membranes can be tailored in terms of their phospholipid compositions, support filter, and type of solvent to successfully predict gastrointestinal or transdermal absorption.

Although many types of in vitro methods are applied during drug discovery and development, the use of cell cultures can be more reliable. Assessment of in vitro absorption, distribution, metabolism, and excretion, as well as drug-drug interaction studies, are mostly performed using various cell culture-based assays. For years, two-dimensional (2D) cell cultures have played a significant role in the testing of various active substances; however, with the appearance of three-dimensional (3D) cell cultures and co-cultures, their importance is decreasing. This is because 3D cultures show protein expression patterns and intercellular junctions, which lead to better IVIVE outcomes as they closely mimic human conditions. Moreover, with the help of co-cultures, it is possible to examine the mutual influence of different cell types [2,8,9].

Compared to in vitro models, ex vivo models are much more complex and therefore closer to human conditions, as these experiments are performed on tissues extracted from humans or animals in a controlled external environment, which allows for higher interplay and cross-talk among the cellular components. They have advantages such as faster and more systematic testing, robustness, and compatibility with high-throughput processes [10]. Therefore, we can consider these models as the tradeoff between in vitro and in vivo methods.

## 2. Oral Route

### 2.1. In Vitro Methods

When discussing the oral bioavailability of drugs, basic factors such as key characteristics of the chemical substance and the dosage form should be considered. The solubility and permeability of drug substances were selected to serve as the basis of the Biopharmaceutics Classification System. The solubility of the active ingredient in the digestive juices should be determined, and the affecting factors must be revealed. Following the suggested strategies of the licensing drug authorities, different buffer solutions are recommended. Buffer solutions mimic the environment of the GI tract; therefore, the pH range of 1.2–6.8 at 37 ± 1 °C is used in most cases. Recent guidelines recommend evaluating drug solubility in buffers at pH 1.2, 4.5, and 6.8. However, several factors could affect the outcome of the solubility investigations. Firstly, the nature of the crystallinity of the substance; compared to the apparent solubility of the crystalline material, amorphous materials possess an increased solubility [11]. Lacking order in their 3D structure, these solid particles form a supersaturated solution; therefore, the drug’s rate and extent of dissolution are increased [12]. The ratio of dissolved drug to total drug content is also important because permeation through the enterocytes’ membrane is aided when the API is molecularly dispersed. On the other hand, supersaturated solutions are thermodynamically unstable. Several factors in the GI environment could contribute to crystallization, but micelles of bile and polymeric excipients could stabilize the solution [13].

Pharmacopoeias contain detailed guidelines to standardize dissolution studies. The purposes of the dissolution test are performed to serve as drug development and quality assurance tools or to provide evidence for adequate similarity or bioequivalence. Pharmacopeial methods and apparatuses must be chosen, but only in cases where modifications are necessary to reveal minor differences in the formulation or the production. Extensive research was initiated to develop biorelevant dissolution tests and biorelevant media. It is well known that simulated gastric and intestinal fluids contain components such as pepsin or pancreas powder at concentrations that are non-physiologically relevant. Volumes and agitation rate rather provide sink conditions and appropriate mixing of the dissolution media than a biorelevant, dynamic environment. Firstly, the media selection should be considered. Empty and fed states of the stomach are good examples to develop biorelevant dissolution fluids. Everyday life habits were summarized when the Fasted-State Simulated Gastric Fluid (FaSSGF) was published [14]. This dissolution media (pH 1.6) mimics the basal, average gastric juice (including sodium taurocholate and lecithin) plus the so-called glass of water used to swallow the dosage form [15]. Consumed food is temporarily stored and digested in the stomach before being passed in smaller portions. It is evident that the artificial fed-stomach media required a more complex composition. Fed-State Simulated Gastric Fluid (FeSSGF) is an acetic acid/sodium acetate buffer with a pH of 5.0. The ionic strength is set with sodium chloride, and the buffer is mixed in a 1:1 ratio with full fat (3.5%) UHT milk as the food part [16]. Along with the gastric media, their intestinal counterparts were created. It is quite interesting that the Fasted-State Simulated Intestinal Fluid (FaSSIF) and the Fed-State Simulated Intestinal Fluid (FeSSIF) were published earlier [17], but their scientific revision and update resulted in a novel version. The second versions of the FaSSIF and FeSSIF are maleate buffers with pH values of 6.5 and 5.8, respectively. The FaSSIF-V2 contains sodium taurocholate and lecithin as surfactants, while the fed-state media is supplemented with glyceryl monooleate and sodium oleate. These two components were meant to mimic the fatty components of the digested food [15].

The digestive and enzymatic functions of the digestive tracts cannot be skipped when improved bioavailability of the formulation is studied [18]. Enzymatic degradation of lipid-based formulations is often mentioned in publications as lipolysis tests. In vitro lipolysis tests are carried out by adding lipid-based formulations (LBFs) to aqueous media (similar to FaSSIF) containing pancreas powder [19,20,21].

It is logical to assume that such formulations, delivering the drug in its lipid-based carrier, are preferred to increase bioavailability. However, when the lipid formulation undergoes enzymatic degradation, drug precipitation may occur, resulting in a negligible effect on drug absorption [22,23]. Enzymatic degradation might be due to colonic fermentation as well. Drugs such as polyphenols possess low upper GI tract absorption, and these xenobiotics could accumulate in the colon, where bacteria metabolize them [21]. Therefore, no consistent data is available to establish a rock-hard correlation, especially in vitro and in vivo correlation [24].

Precipitation is also an important factor, not only in the digestion or degradation of the formulation but also when it is transferred from one digestive compartment to another. The pH-dependent solubility of the drug is also important when such transfer dissolution tests are performed. A basic setup is when two compartments of paddle-type dissolution apparatuses are connected with a peristaltic pump. The donor compartment is acidic (pH 1.2–2.0) with a smaller volume compared to the acceptor or intestinal compartment, where the buffer is almost neutral (pH 5.0–6.5). The transfer rates are varied, and zero- or first-order kinetic approaches were used. In the case of the zero-order kinetic transfer model, a constant volume is pumped, while in the first-order model, the transfer speed (mL/min) decreases over time. These experiments were used to successfully describe the precipitation rates of marketed formulations of drugs with pH-dependent solubility [25,26].

The previously detailed dissolution studies (with biorelevant and transfer experiments) could be integrated into systems where the permeability of active ingredients could be measured. In vitro drug permeability is often screened in parallel artificial membrane permeability assays (PAMPA). PAMPA assays are high-throughput experiments predicting the passive transcellular permeability of active ingredients. Biomimetic membranes, separating the donor and acceptor phases, can be tailored regarding their phospholipid compositions, support filters, and type of organic solvent [27,28]. Combined in vitro dissolution and membrane transport experiments were carried out in a modified paddle apparatus where the absorption chamber was submerged into the dissolution vessel. The dissolution compartment or donor compartment was separated by the hydrophobic membrane, resulting in an integrated in vitro dissolution-absorption system where the temperature was maintained at 37 °C and drug concentrations in both chambers were detected using fiber optic probes. In Pion’s MacroFLUX™ system, the hydrophobic membrane was made by placing n-dodecane or 20% lecithin dissolved in n-dodecane on the hydrophobic polyvinylidene fluoride filter. To mimic the pH changes during indigestion of the drug formulation, the initial acidic solution (artificial gastric fluid) was converted to FaSSIF. This method was successfully used to detect pH-limited drug dissolution and its effect on the membrane flux. On the other hand, the effect of excipients was revealed among different marketed drug products in terms of drug absorption [29,30].

### 2.2. Cell Culture

**Figure 1:**
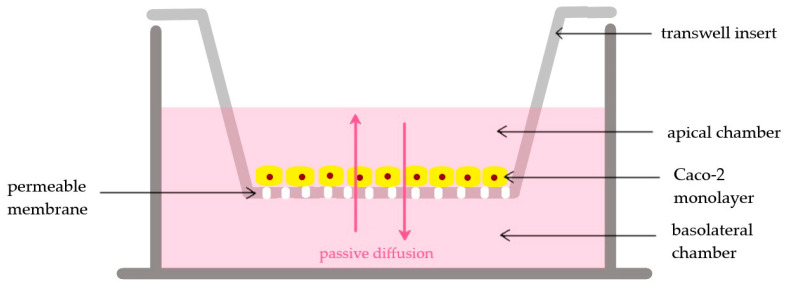
Schematic diagram of a conventional Transwell plate with the Caco-2 cell monolayer for the investigation of oral bioavailability.

Studying the biorelevant permeability of the drug or formulation requires cell lines, namely the Caco-2 cell culture model, besides artificial membrane assays. Originally, cells were isolated from a human colon adenocarcinoma [31]. They were proven to be an excellent cell culture model for investigating the cellular uptake or the transepithelial permeability through the monolayer they form [32]. When cultivated under certain conditions, they polarize and form monolayer-expressing receptors of the human enterocytes, thus mimicking the absorptive properties of the small intestine [33]. In vivo, the polarized enterocytes face toward the intestinal lumen with their apical surface. This surface is directly exposed to the content of the intestinal lumen [34], and the contact is enormously increased due to the presence of the microvilli [32]. The standard method used to differentiate Caco-2 cells is to seed them on a microporous surface, such as polycarbonate cell culture inserts. On the supporting surface, the cells grow and form a monolayer with tight junctions among the neighboring cells; therefore, an apical and basal compartment is created (Figure 1) [35,36]. Permeability or transport studies are performed by adding the drug or formulation to the apical chamber and detecting its concentration in the basal chamber. The integrity of the monolayer is a vital factor for transport studies, especially when the passive transport of drug or formulation through tight junctions can be expected. To check the high confluency, besides confocal microscopy, fluorescently-labeled markers such as fluorescein [33,37], lucifer yellow [38], or radiolabeled markers like C^14^ mannitol [24] are used. Additionally, the transepithelial electrical resistance (TEER) should be monitored on a regular basis using a volt-ohmmeter equipped with a “chopstick” electrode [38,39]. The integrity of the Caco-2 monolayer can be considered sufficient if the TEER values are at least 800–900 Ω cm^2^ [35,36]. This must be checked before and after the transport experiment well by well [38]. A sudden drop in the TEER values indicates a breakdown in the cellular barrier integrity [40].

Transport experiments can be performed not only with the Caco-2 cells; intracellular uptake or drug internalization could be investigated by fluorescent microscopy or flow cytometry as well. These studies are also important to reveal a possible transcellular pathway of drug absorption [40,41]. The in vitro cytotoxicity or cell viability assays are also extensively used and are cost-effective methods to screen excipients to ensure biocompatibility in their applied concentration. Simple colorimetric assays, such as MTT or neutral red assays, are used to measure the viability of the Caco-2 cells [42,43]. On the other hand, the kinetics of epithelial cell reaction to excipients or formulations can be monitored by impedance measurement with a real-time cell analyzer. This method is non-invasive and label-free, and it linearly correlates with the growth, adherence, and viability of cells [38].

Since cultivated cells are living systems, there is variability in the reproducibility and stability of these models. As an example, decreased TEER values have been detected over time. This problem often extends to cell culture studies and could cause an increase in expenses. Additionally, the transporter proteins are expressed at a different level than in vivo and tiny changes in the cell culture media have a considerable effect on the cell’s phenotype. Unfortunately, the results obtained in the Caco-2 cell model may vary among different laboratories [32,37].

### 2.3. Ex Vivo

Ex vivo methods provide a theoretical means of estimating absorption and bioavailability. They include three main methods: diffusion chambers with separated tissue, everted gut sac, and intestinal perfusion. Ex vivo models have adequate paracellular permeability, mucus layer [44], transport protein expression [45], microbiome [10], and metabolizing properties [46] that separate them from the in vitro Caco-2 model. Ex vivo methods are simple and widely used in the design and testing of potential new drugs or new formulations [47,48].

Diffusion cells can be divided into two major methods, considering the used tissue. In the case of the Ussing chamber, mainly animal or possibly human biopsy intestinal samples are used for the transport model, and the intestinal segment is placed between the two compartments [49,50]. The physiological medium (Krebs, Ringer, PBS, Hank’s solution) [51] is circulated separately in both compartments, and the needed gases are ensured by bubbling carbogen gas. The model is suitable to investigate mouse, rat, rabbit, dog, rat, and monkey tissues, which show a very good correlation with the human in vivo results [50]. Furthermore, the method can be used to study differential absorption in pediatrics [52] and to investigate the absorption windows [53]. There are many references in which ex vivo results are well correlated with in vivo results [54,55,56,57,58], but this method requires very expensive equipment, and we cannot ignore the fact that the tissue maintains its integrity for only 2–3 h [5].

Franz cells are similar to the Ussing chamber, but the tissue sections are placed horizontally compared to the above-mentioned method. This diffusion cell model was used for just buccal permeation studies in the oral route because it has limitations for intestinal permeability due to the uncontrolled donor temperature [5]. The continuous mixing effect can lead to higher permeation as observed for standard permeability markers than in the case of the Ussing chamber; therefore, it is beneficial for examining thicker tissues such as skin, avoiding tissue damage [59]. During oral drug administration, the first anatomical site is the buccal tissue, where the active substance can be absorbed. The Franz diffusion cell is most suitable for examining this ex vivo absorption [60,61]. The dog buccal mucosa is similar to human morphology and immunohistology; it shows high drug permeability with moderate correlation with the human study [62,63]. The most common tissue type is porcine, which shows a high correlation; meanwhile, the cost is low and easy to obtain [64]. The thickness is large, so the permeability is low [65]. The rat tissue can be obtained easily at a low cost; nevertheless, due to keratinization, it is not widespread and less correlated with the human surface [61,66]. It is also possible to use rabbit, monkey, or chicken tissue, but these are not very common, either due to the difficulty of obtaining and handling them or the size of the tissue.

The everted rat and hamster intestinal sac models were published first by Wilson and Wiseman [67]. The intestinal sections (duodenum, jejunum, ileum) are cut into small tubes and everted. The mucosal surface is opened towards the API-containing buffer solution, and the serosal layer forms the inside of the sac, which is filled with buffers and solubilizing agents (SLS, Macrogol, Tween). In the case of careful handling of the extracted tissues, the tissue life can be extended up to 2 h from 30 min, which makes it suitable for examinations. Proper handling of the tissue includes keeping it on ice until the test begins and bubbling carbogen gas in the buffers [5,47,68,69]. Standard molecules (mannitol, antipyrine, and digoxin) showed excellent correlation with the everted sac model [70].

The flow through cells can be used to perfuse the API-containing medium through the evacuated intestinal segment, allowing the study of uptake in several intestinal segments simultaneously. The method involves circulating the drug solution in the intestinal segments while measuring the change in concentration. In this case, the media described above and an adequate gas supply are also necessary for the tissue to survive. Although the method is not widespread because of its difficulty, it shows reproducible results because several samples are tested under the same conditions [71].

In Table 1, we have summarized the recent regulatory statuses of the above-mentioned techniques or methods when the bioavailability assessments are performed.

## 3. Nasal or Inhalation

### 3.1. In Vitro

Nasal and inhalation drug delivery has become a common administration route in the last decades for local and even systemic therapies, and there is a growing interest in developing new formulations as well. For these drug delivery routes, the main challenge is to characterize the nasal and/or lung deposition pattern in vivo [77]. Although the recommended in vitro tests, for example, particle size distribution, spray pattern, or emitted dose, are useful to characterize or compare nasal dosage forms and ensure the required quality of the product, they provide limited information about nasal deposition, pharmacokinetics, or pharmacodynamics [78]. Nasal casts offer a cost-effective and rapid method for addressing this issue before the beginning of complex in vivo studies. Even though studies with nasal casts are not a regulatory requirement, the results may provide beneficial information for further development. Targeting the deposition in the nasal cavity has a great impact on the efficiency of local, systemic, and central nervous system drug delivery as well. With the help of anatomically correct in vitro nasal models, the deposition pattern of different nasal preparations can be compared [79]. First nasal casts have evolved from cadavers’ heads as they strongly represent human nasal anatomy. To avoid the limitations of tissue preservation issues, the water and lipids were replaced by silicone plastination, resulting in a much more representative structure [80]. Recently, nasal casts are mainly obtained from computed tomography scans with the help of 3D printing since this technology has gained great interest. Most commonly, they are made of plastic, silicone, resin, and acrylonitrile butadiene styrene (ABS) [81,82,83]. Depending on the specific nasal pathway targeted, the cast model can be divided into 2–3 or 5–7 anatomically relevant regions. Shah et al. designed a seven-section nylon nasal cast according to the computed tomography images of healthy humans. The casts were coated with a glycerol/Brij-35 solution in order to mimic the mucus [84]. Hartigan et al. designed a 3D-printed in vitro tissue model using ABS filament with a fused deposition modeling 3D printer for preclinical validation of experimental swabs. To mimic the soft human tissue of the nasal cavity, they used aqueous silk sponges; thus, it does not require any cellular material, and the physiological nasal fluid was replaced with synthetic mucus. This model may provide a reproducible, safe, and cost-effective tool for the development of newly designed devices [85]. In order to obtain further information about biological aspects such as permeation, mucoadhesion, and ciliary clearance, in vitro anatomical models can be used in combination with both primary and immortalized cell cultures as well [86].

**Figure 2:**
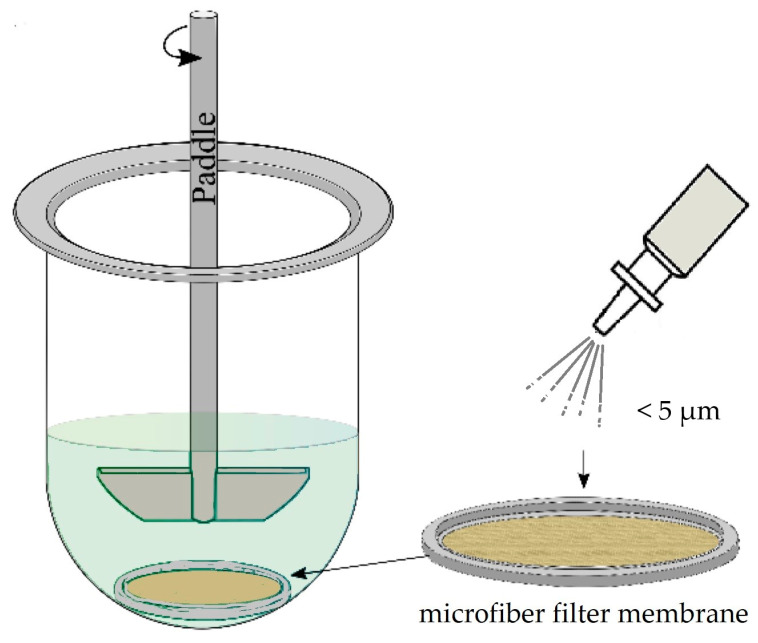
Schematic of paddle-over-disc USP 5 apparatus with a microfiber membrane accumulated with powder mass for the investigation of nasal drug delivery.

As for inhalation drug delivery, particle size and in vitro dissolution are the main in vitro parameters that determine the bioavailability of inhaled drugs. The most important aspect that must be taken into consideration is that only a fine fraction of the aerosol (particles <5 μm) reaches the deep lung, and thus, is available for in vitro dissolution testing. This means that the commonly used dissolution protocols for oral dosage forms require adaptations. Mixing forces in the lung are minimal; thus, agitation is questionable during dissolution. As the temperature in the respiratory tract is usually lower than that in the gastrointestinal tract, the testing temperature should be 32.5–33.5 °C according to measurements in healthy volunteers [87]. Since there are no available guidelines provided by regulatory agencies, several dissolution protocols have been described in the literature and developed by researchers. With the help of the UniDose aerosol collection system, the whole powder mass accumulates on a glass microfiber filter membrane and is placed into a disk cassette in the USP2 apparatus (Figure 2). Other methods disperse particles in phosphate-buffered saline, a salt solution with a similar composition to the human hypophysis, or PBS with 0.1% Tween 80. Reproducible and stable physiological dissolution fluids are yet to be identified and standardized. USP1, USP2, USP4, transwells, and Franz diffusion cell systems are all used to expose solid particles to the dissolution fluid, as there is still no adequate in vitro exposure system that can mimic dissolution in vivo [88].

### 3.2. Cell Culture

The respiratory tract is promising as an alternate site of drug delivery because of fast absorption and quick onset of drug action; by avoiding the first-pass metabolism, it is highly beneficial compared to the oral route. Currently, the pharmaceutical industry extensively relies on suitable in vitro models for the faster evaluation of drug absorption and metabolism as an alternative to animal testing. In vitro cell culture models of the respiratory system can decrease the development time for new medicinal products. In the pre-clinical phase, they can be utilized as an alternative to pricey and time-consuming animal testing. Consequently, the information on absorption mechanisms gained from in vitro studies allows for the directed and cost-effective performance of clinical studies and the safety evaluation of new active substances and chemicals [89].

Primary cells and cell lines differ from each other by the time they can be kept in culture. Primary cells must be freshly isolated; thus, they originate from a different individual each time. Primary cultures have some significant limitations, such as the difficult accessibility of suitable human airway tissue and the limited number of cells. On the other hand, they provide the closest in vitro representation of the epithelium [90].

Immortalized cell lines are derived from different tumors, and their advantages over primary cultures are the purity of the cell types and their unlimited lifespan, which enables prolonged experiments. These cell lines are easy to maintain, and they exclude many difficulties, e.g., reproducibility or high costs. The epithelial cells coating the respiratory system play an important role in the protection of the host from different stimuli, including chemicals and pathogens. Human airway epithelial cell cultures are essential for studying aspects of respiratory tract biology, disease, and therapy [91].

Immortalization can cause the occurrence of undesired morphological changes in the cells. Nowadays, the only immortalized nasal cell line of human origin is the RPMI 2650.

RPMI 2650 cells were first isolated from anaplastic squamous cell carcinoma in the nasal septum. The main differences between the nasal mucosa are the absence of ciliary movements and the multilayer cell growth. Despite these few differences, RPMI 2650 cells have shown a similar permeability to nasal mucosa for hydrophilic, lipophilic, and high molecular weight compounds. To perform transport studies, the tight junctions and cell monolayer are crucial points. Furthermore, the cells should be in a high differentiation state. As RPMI 2650 cells are not able to form a monolayer and they lack tight junctions, they are not suitable for transport studies. However, they can be co-cultured with fibroblasts or endothelial cells, which can form a confluent monolayer and develop tight junctions as well. Thus, RPMI 2650 in a co-culture can be a reliable in vitro method for nasal drug absorption [92,93,94].

16HBE14o- cells are normal human airway epithelial cell lines. They can form polarized cell monolayers and display many properties of bronchial cells, e.g., showing lectin-binding patterns and expressing the intercellular adhesion molecule. The confluent monolayers show extensive tight junctions, which are similar in appearance to those exhibited in intact human tissue. A great disadvantage of the cell line is that it does not secrete mucus that protects the epithelium in vivo, in contrast to Calu-3. 16HBE14o- cell line is assessed with great potential as a bronchial drug absorption model [95,96].

Calu-3 is a human sub-bronchial gland cell line; it was derived from bronchial adenocarcinoma. Calu-3 expresses mRNA and proteins specific to the native epithelium. The cells can form confluent monolayers with tight junctions; the cell line can be an appropriate tool for studying tight junction regulation in the bronchial epithelium [89]. Due to their origin, Calu-3 cells can produce mucus as well. The presence of tight junctions and the secretory activity highlight the potential of this cell line as a suitable in vitro model for studying the pulmonary drug absorption [97,98].

The in vitro cell culture modeling of the respiratory system is not an easy process, but in recent years several solutions have been developed to address the problem. It is crucial to take into consideration the characteristics and limitations of the selected cell culture when designing the experiment and to ensure that the cells are suitable. It is important to compare the properties of the selected cell line with those of the relevant intact tissue, as it is demonstrated above that cell cultures may have shortcomings. For the respiratory system’s cell cultures, such important factors may include mucosal secretion, modeling of ciliary activity, the presence of tight junctions, protein expression, or monolayer formation. Primary cells are a lot similar to intact tissue; however, they have several limitations as well.

### 3.3. Ex Vivo

The isolated and perfused lung model (IPL) was developed and used in the physiological investigation to advance lung transplantation. However, in the last 20 years, it has been used in the investigation of the absorption of inhaled drugs [99,100].

Another method to investigate lung permeability is the precision-cut lung (PCL) method, which truly represents the lung’s structural and functional cellular interaction. However, the disadvantages of the method include its inability to mimic ventilation, mechanical stretch, and perfusion [101,102,103]. To determine the in vivo toxicities, drug permeabilities, and therapeutic efficacies in ex vivo, the PCL model is clinically relevant [104]. Table 2 serves as a representation of the current regulatory acceptance statuses for the in vitro methods and ex vivo opportunities used for evaluating the bioavailability of drugs after intranasal or pulmonary administration.

## 4. Transdermal

### 4.1. In Vitro

The dissolution test of semi-solid preparations is a constantly controversial topic because none of the pharmacopoeias have an appropriate section on the dissolution test without a membrane. The Pharmacopoeia recommends three different methods only for patch testing, namely disc assembly (Ph. Eur. 2.9.4.1./USP Apparatus 5), cell (Ph. Eur. 2.9.4.2), and rotating cylinder (Ph. Eur. 2.9.4.3/USP Apparatus 6) [107]. In each case, the active substance is released freely, and the patches are fixed with a 125-mesh net, which keeps the preparation in place and does not affect the way of diffusion. These dissolution studies can be used to compare preparations, but less so for true in vivo correlation [108,109,110,111].

In the literature, there are various in vitro release and penetration studies, that can be used to predict in vivo bioavailability [112]. In vitro penetration studies usually use some type of diffusion cell, where passive diffusion is tested on various types of synthetic membranes [113]. The flowthrough cell, vertical diffusion cell, and immersion cell methods were published to describe the dissolution profiles of formulations; nevertheless, the vertical diffusion cell method is the most widely accepted.

**Figure 3:**
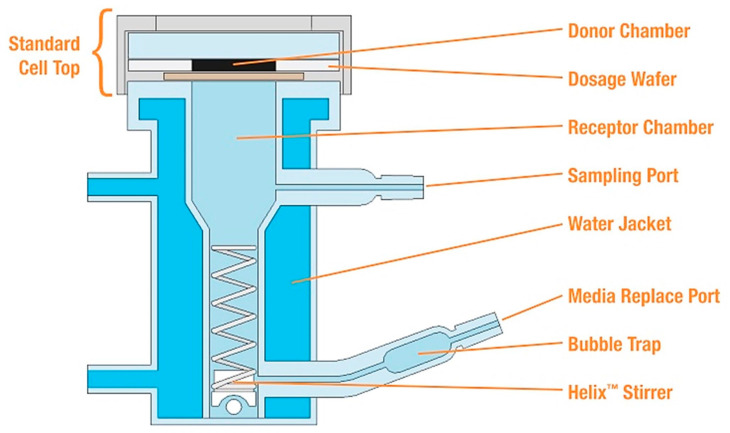
Schematic of a vertical Franz-type in vitro diffusion cell.

The vertical diffusion cells include the same basic parts as a donor and an acceptor compartment and a membrane between the two compartments (Figure 3), but in the literature, a different semantic structure is available to avoid the entrapping bubble or minimize human intervention [114,115,116]. The acceptor compartment usually contains a buffer and a magnetic stirrer at the bottom of the device, which helps to ensure homogeneous distribution. At various intervals, the acceptor phase is sampled and replaced with a fresh acceptor solution. The limitation of the method and its in vivo correlation are determined by the acceptor buffer and the used membranes. The Start-M membrane and the Tuffryn membrane show a good correlation with in vivo results. The Strat-M membrane is a synthetic, non-animal-based model membrane made from polyether sulfone and polyolefin, which are predictive of diffusion in human skin. The Tuffryn membrane is a polysulfone membrane with low protein binding; hence, it is suitable for the permeation study of biological markers. In most cases, they are used with a buffer and solubilizer agent, such as SLS, Volpo, Tween 80, and PEG 10000 [117,118,119,120].

A horizontal diffusion cell, also known as a side-by-side diffusion cell [121], also consists of two compartments, but they are smaller in volume and have a smaller diffusion area than vertical cells. Its use is appropriate for small quantities of materials, and it also eliminates the shortcomings of vertical systems, such as the fact that the donor phase is not heated. However, their use is nowadays declining.

The flow-through diffusion cell works similarly to the diffusion cells described above, but with this device, the sampling can be automated [119], as a fraction can be collected from the flowing acceptor phase [122]. Moreover, by setting the parameters of this device, such as the acceptor volume and flow rate, better in vitro-in vivo correlation can be achieved. An important aspect of these measurements is the in vitro-in vivo correlation, which is a new method that has been developed nowadays. This is a bio-predictive IVPT method using a flow-through diffusion cell with the Strat-M membrane mentioned above [117].

Nowadays, a wide range of complex membrane systems is available to simulate human skin. Based on the aforementioned parallel artificial membrane permeability assay (PAMPA), Ottaviani et al. were the first to publish the use of a skin PAMPA. In this case, the membrane was filled with an optimized mixture of silicone (70%) and isopropyl myristate (30%) to reduce skin permeability, thus making it even more similar to human skin [7,123,124].

Lately, a new skin PAMPA model has become available, where the membrane contains special components of the skin barrier, such as cholesterol, free fatty acids, and ceramides that mimic the properties of the lipid matrix [125]. Another advantage is that this skin PAMPA is a 96-well plate-based method, so it can be a relatively quick and low-cost model and an effective high-throughput assessment technique [7].

### 4.2. Cell Culture

The skin tissue is an effective barrier, representing a protective layer and an essential interface between the human body and the external environment. Based on its structure, it may affect the topical and transdermal bioavailability of various substances. Dermal absorption studies are routinely used to demonstrate benefits after topical application of cosmetics, pharmaceutical formulations containing active ingredients, transdermal patches, or medical devices, but also to predict risks from skin exposure to chemicals [126]. To receive reproducible data on percutaneous absorption, there is an increasing demand for reliable in vitro models, as the national legislation lays down that animal experiments should be avoided whenever scientifically feasible. In addition, the results of animal experiments do not always correlate with the results of human clinical studies due to differences in the structure of the skin [127]. Regarding human and animal models, the skin is associated with other organs, making it difficult to characterize skin diseases independently [128]. Furthermore, a standardized in vivo human or animal skin model is not yet available. Internationally accepted guidelines were created by the Organization for Economic Co-operation and Development (OECD), which give specifications for testing the in vitro percutaneous absorption of chemicals [129].

In recent years, several in vitro skin models have been developed using different cell cultures to assess the penetration and permeation profiles of active ingredients. The oldest but most used methods for constructing skin models are mainly the primary cells, for example, epidermal cells (keratinocytes and melanocytes) or dermal cells (fibroblasts and human dermal microvascular endothelial cells), and cell lines such as the immortalized human keratinocyte cell line (HaCaT), the human foreskin fibroblast cell line (HFF-1), and the murine NIH3T3 fibroblast cell line [130]. The biggest concern is that the primary cells and cell lines do not necessarily represent what happens in vivo, as the cell-cell and cell-matrix interactions, the diversity of the cells that make up the skin (e.g., melanocytes, Langerhans cells, and endothelial cells), and skin appendages (e.g., sweat glands and hair follicles) are missing. The cells do not grow on top of each other but are forced into a monolayer morphology, which is unnatural for most cell types. Despite these disadvantages, they are the most accurate methods of establishing scientific results for long-term research projects [131,132].

In vitro skin models, such as 2D monolayers of human skin cells created by tissue engineering, have shown the possibility of a more accurate, systematic characterization of the skin [128,130]. Establishing co-cultures (e.g., co-cultures of keratinocytes with immune cells and dermal fibroblasts) on Petri dishes or microtiter plates can increase natural intercellular contact and communication, but the 2D surface still inhibits the capacity for cells to form a multi-dimensional structure, which is limiting their accuracy in predicting the complicated effect of drug metabolism on the skin [133]. Therefore, cells grown in flat layers on plastic surfaces do not accurately model in vivo cells.

As a solution to the above-mentioned problems, many 3D models were developed in the form of reconstructed human epidermis (RHE) generated by seeding keratinocytes on a porous membrane. With the creation of these systems, the common goal was to bridge the gap between the use of animals and cellular monolayers [134].

Several RHEs are commercially available that exhibit actual similarities to the native human tissue in terms of morphology, biochemical markers, and lipid composition. These systems are appropriate devices for the testing of phototoxicity, corrosivity, and irritancy caused by different substances; moreover, transport studies can also be carried out. The EpiSkin™ (L’Oréal, Lyon, France), EpiDerm™ (MatTek Corporation, Ashland, MA, USA), SkinEthic™ (Lyon, France), and EpiCS^®^ (CellSystems, Troisdorf, Germany) skin models are well documented in the literature [135,136]. EpiSkin™ and EpiDerm™ were the first 3D models developed and validated as predictive models for skin corrosion and skin irritation [137]. These two models contain human keratinocytes cultured on a collagen-based matrix, simulating the in vivo skin epidermis. Other models, such as SkinEthic^®^ and the modified EpiDerm™ SIT, are used to test irritation on the skin [137,138,139].

Dreher et al. investigated the cutaneous bioavailability of formulations containing caffeine or alpha-tocopherol on RHEs (EpiDerm™ and EpiSkin™) and compared them with ex vivo studies conducted on human skin. They found that vehicles that contained alcohol showed more potent drug permeation rates in the case of EpiDerm™ and EpiSkin™ models compared to ex vivo results. This was attributed to the weaker barrier properties and the increased hydration of the outermost layer of the stratum corneum of the RHEs [140]. Similar studies were conducted by Schäfer-Korting et al. to evaluate the permeation of caffeine and testosterone across different RHE models (EpiDerm™, EpiSkin™, and SkinEthic™), human epidermis, and animal skin. The permeation coefficients of testosterone were in the following order: human epidermis, bovine udder skin, porcine skin < EpiDerm™, EpiSkin™ < SkinEthic™, while, for caffeine: bovine udder skin, EpiDerm™, porcine skin, human epidermis < SkinEthic™, EpiSkin™. Moreover, the investigated RHE models were validated by them, using nine different drugs with different physicochemical properties. The permeation rates of all substances were higher through the RHE models compared to the human epidermis and porcine skin; however, the ranking of drugs according to permeability was found to be similar on all membranes. They also found that the reproducibility of permeation parameters was very similar for RHEs and for excised skin [141].

Lotte et al. investigated the reproducibility of three RHEs (EpiDerm™, EpiSkin™, and SkinEthic™) regarding the permeation and skin absorption of topically applied compounds with different physicochemical properties (lauric acid, caffeine, and mannitol). They described that SkinEthic™ showed the worst reproducibility among these three models [142].

Jírová et al. examined the skin irritation of different chemicals with in vivo and in vitro methods using the 4-h human patch test (HPT). It was described that the concordance of human epidermis models with human data was 76% (EpiDerm™) and 70% (EpiSkin™). The sensitivity and accuracy of the irritant classification of the RHEs were higher than expected, and they showed better results compared with the tests conducted on rabbits [143].

These studies concluded that RHEs could be considered as alternatives to human, porcine, or rabbit skin for in vitro studies. However, despite the above-mentioned huge advancements, most of the 3D skin models still have some limitations such as weak barrier function, lack of vasculature and skin appendages, and thus, are not able to fully reproduce the complexity of human skin tissue [132,133,144].

In parallel with the development of RHE models, efforts have been made to add a living dermal compartment to produce models referred to as full-thickness (FT) skin models. These models make it possible to seed keratinocytes directly onto the surface of the formed dermal lattice layer, and thus, allowed the investigation of ultraviolet-A-induced aging [145], skin metabolism, genotoxicity [146], and the role of papillary and reticular fibroblast populations [147], as well as glycation in aging to be deciphered [148].

The development of an FT skin model is beginning with the generation of a mature dermis and with the expansion of melanocytes and keratinocytes on top of the dermis, followed by the development of the epidermis at the air–liquid interface [149].

The commercially available FT skin models such as PhenionFT™ (Henkel, Düsseldorf, Germany) and EpidermFT™ (T (MatTek, Ashland, MA) are widely used in the investigation of environmental and age-dependent effects [150], skin penetration, ultraviolet (UV) irradiation effects, and skin disease mechanisms [151]. A novel FT skin model, T-Skin™, is an in vitro reconstructed skin that consists of a dermal equivalent with human fibroblasts overlaid by a stratified, well-differentiated epidermis derived from normal human keratinocytes cultured on an inert polycarbonate filter. Batallion et al. compared the structure and the layer-specific markers of the T-Skin™ (Episkin, Lyon, France)with normal human skin using histological and immunohistological staining. It was found that T-Skin™ exhibits a very similar structure and characteristics to the human skin, including a well-differentiated and organized epidermis and a functional dermis [152]. These results support the use of T-Skin™, as an alternative screening platform, to develop new cosmetics and to investigate dermatologically active ingredients.

Recently, given the increased interest in extending the experimental testing phase, a long-term FT skin model became commercially available (Phenion^®^ Full-thickness LONG-LIFE skin model, (Henkel, Düsseldorf, Germany), which can be kept in culture for up to 50 days [149].

Significant progress for skin models may be achieved in the field of organ-on-chips, which can provide more physiological conditions with the combination of microsystem engineering and cell/tissue biology [132]. To develop a physiologically relevant in vitro skin model, human skin structures have been integrated into microfluidic systems to construct skin-on-chip models, which can mimic the complex in vivo situation [133]. Microfluidic technology makes it possible to develop an ideal skin-on-chip model by being able to create specific flow properties to ensure efficient chemical reactions, thus the cell-cell and cell-matrix interactions can work properly. Microfluidic-based organ-on-chip systems consist of microchannels, micropumps, valves, mixers, and integrated biosensors, with cell culture inserts used to develop in vitro functional models of healthy and diseased organs [128,153]. Using these devices, the skin tissue can be cultured under the control of several physical and biochemical parameters, such as flow, force, or chemical gradients [133,154].

The fabrication process, materials, and tissue maintenance of these in vitro models can vary greatly. There are two main groups to which the skin-on-chip models can be classified according to how the skin is generated in the chip: the first one is the direct transfer of a skin fragment from a biopsy or a human skin equivalent in the chip (transferred skin-on-a-chip), while the second one is based on the in situ generation of the tissue directly on the chip (in situ skin-on-a-chip) [154,155,156].

However, there would be some limitations in verifying the correct skin differentiation and structure. To overcome this problem, most of the researchers are using fluorescent-labeled cells or traditional immunocytochemistry for visual inspection, but in most cases, the detection process can be very complicated. This fact led to the development of biosensors which can be completely integrated into the chip to follow up the state of the skin in real time and to monitor the effects of the applied active agents [154].

Overall, skin-on-chip models could be the best platform to study intercellular interactions or even the immune response, as they can better reproduce the physiological environment of the tissue. Furthermore, they allow us to follow up several conditions at the same time under controlled parameters and measure drug efficiency rapidly.

### 4.3. Ex Vivo

The Franz diffusion cell is the most widely used ex vivo model for evaluating the release and skin permeation of API from topical and transdermal drug delivery systems. The main aim of these studies is to identify the main potential variables that may alter the in vivo bioavailability of the drug during formulation design [59]. The design and principle of operation of the test apparatus correspond to that described for the buccal ex vivo section. Several skin preparations were used, such as mouse [116], rat [157,158] porcine ear [159], newborn pig skin [160], or human skin [161]. Ears of porcine are used mostly because they are easily obtained and cheap; furthermore, the in vivo correlation is also good [120,162,163].

Evaluation of the penetration of active substances in the skin is essential for developing topical formulations, as the expected effect remains on the skin’s surface. The concentration of the active ingredient in the skin layers (stratum corneum, epidermis, and dermis) can be determined by ex vivo and in vivo studies by skin retention tests [59]. After the Franz permeability test, the skin can be dried and separated into three main layers. Cellophane tapes can be used 25 times to remove the stratum corneum. The remaining epidermis and dermis can be divided into two parts by heating (60 °C) and mechanical. The API extractions can be carried out in methanol and using an ultrasonic bath at 40 °C for 15 min [164].

Information on the distribution of the active substance within the skin can be easily achieved using RAMAN spectroscopy. The Raman correlation map shows the incidence of the API in the different layers of the animal or human skin from the stratum corneum to the lower layer part of the epidermis. The Raman experiments give a good correlation with the Franz cell and skin PAMPA results, which, thus, closely approximate the in vivo results [7]. Techniques and methodologies used for the evaluation of transdermal bioavailability of drug formulation are summarized in Table 3 regarding their regulatory status.

## 5. Ophthalmic

### 5.1. In Vitro

The unique anatomical structures of the eye represent multiple barriers, which modify drug absorption [168]. This and the different fluid dynamics make this organ an especially hard-to-simulate environment to develop in vitro, ex vivo, or cell culture techniques for bioavailability testing [169]. No true, validated method is available for the replacement of the in vivo Draize rabbit eye test, which is the gold standard for not only bioavailability but also toxicity testing due to its high similarity and ease to use compared to other mammalian models [170].

In the case of some APIs, FDA guidelines allow the use of bioequivalence testing according to 21 CFR part 320 with different in vitro methods for ophthalmic products instead of a human clinical end-point study in case of a special condition [171,172]. This is the qualitative (Q1) and quantitative (Q2) sameness of the test and the reference product, enabling a ± 5% concentration difference in the case of inactive ingredients. Physicochemical characterization including measurement of pH, osmolality, viscosity, particle size distribution and charge (for emulsions and suspensions), phase distribution, specific gravity, and surface tension measurement is needed [173,174]. In vitro drug release tests can be carried out with the use of dialysis tubes and Franz diffusion cells [175]. These experiments are usually carried out in either artificial tear fluid or normal PBS solution with membranes and a dialysis bag made out of cellophane or some cellulose derivative with a molecular weight cut off around 10 kD [176,177,178,179].

### 5.2. Cell Culture

2D and 3D cell culture models vary in form and the utilized cell line. In general, they are cheap, easy to maintain, and reproducible ways to carry out drug transport experiments. Meanwhile, the modern but complicated in vitro 3D cell culture methods, such as the reconstructed human tissue assays SkinEthic™ Human Corneal Epithelium and EpiOcular Eye Irritation test, are rarely utilized by current researchers due to their high price, low repeatability, and limited transition towards in vivo results [169,180,181]. Apart from these, 2D cell cultures of immortalized cell lines such as ARPE-19 or TERT-RPE are mainly used to screen cytotoxicity before in vivo tests. Nevertheless, cell cultures can be useful tools to verify cellular uptake of the API before an animal experiment. Simple, 2D cultures ARPE-19 cells were successful in the study of Yang et al. in regards to the prediction of cellular retention of bovine serum albumin-loaded silk fibroin nanoparticles when compared to in vivo rabbit eye model [182]. In the case of atorvastatin-loaded solid nanoparticles, the same correlation can also be seen as the cells showed high levels of retention of the API [183]. Even the similarity of ocular PK values for melanin binding was found between ARPE-19 cells and rat eye model [184]. Human corneal epithelial cells in 2D cultures were also reported as good indicators of the resveratrol uptake [185]. Despite these positive examples in the study of Yousry et al., normal human primary corneal epithelial cell lines failed to predict the superior uptake of a terconazole SNES over simple suspension, which was later verified by animal experiments [186].

### 5.3. Ex Vivo

An upgraded version of the previously mentioned permeability test is when excised animal corneas are used as the “membrane” of a Franz diffusion cell, using artificial tear fluid as solvent [187,188,189,190]. Apart from corneas, even whole eyeballs can be used [191]. Overall, these methods are not officially validated, but the in vitro and ex vivo results are usually used to reduce the number of experimental formulations selected for the in vivo experiments. Thus, these methods usually act as a filter for the multiple original formulations with different excipients, as only those get a pass on to the animal model, which has the best pharmacokinetic/dissolution profiles.

Notably, apart from the traditional Draize test, the implantation of a microdialysis probe into the anterior segment of the eye gives the researchers the ability to test out multiple concentrations of an experimental formulation and gain additional dissolution profiles [190,192,193].

The regulatory acceptance and opinion are summarized in Table 4.

## 6. Conclusions

Summarizing the methods and techniques to assess the drug availability during drug R&D or formulation development phases is essential for screening for optimal molecules and their dosage forms. When the bioavailability of drugs administered via oral, transdermal, intranasal/pulmonary, or ocular pathways is assessed, in vitro screening tests, due to their low cost and high throughput performance, are inevitable. Designing the in vitro tests, either for solubility assessment of the solid dispersion or the deposition of the fine particles, is all based on those physiological factors that are vital and responsible for effective drug therapy. Biopharmaceutical drug design also relies on the interaction among the drug, the carrier or dosage form, and the first biological barrier determining absorption. Cell culture models, either primary or immortalized cell lines, provide the first biorelevant permeability data, even with drawbacks such as different transporter or junctional protein expression. Dermal and ocular barriers are more complex compared to the intestinal, pulmonary, or nasal barriers. Modeling the complexity of such cellular barriers can be integrated into simplified 3D cell culture models. Due to their complexity, they are more biorelevant when drug permeability is investigated. In the case of RHE, a comparison of the drug with the standards could predict in vivo permeability rates. On the other hand, when passive diffusional pathways are considered in such cases, the actual in vivo data might not correlate with the data obtained in such models. The last step before investigating the drug or its formulation in vivo is to obtain data on human or animal tissues. Besides considering the ethical problems related to the origin and source, differences among the species are another issue researchers should not forget. Due to their limited lifetime, excised tissues or organs require experienced human resources and device setups. Expertise in these studies can be obtained in laboratories, where the surgical and analytical background is provided and validated. Considering the source of the excised tissues or organs, ethical questions almost immediately appear in our focus. All the efforts made to replace lab animals, reduce their number during preclinical studies, and refine study protocols and statistics in data analysis cannot completely predict the in vivo performance of the formulation. Regulatory authorities did not integrate artificial membrane studies into their guidelines when drug permeability is evaluated in vitro, except for transdermal dosage forms. Additionally, the Caco-2 cell culture model is only recommended by the FDA; thus, incomplete harmonization is present among the agencies. Still, a gap exists among the phases of drug development and characterization, starting from the in vitro evaluation through the cell culture laboratories and the absorption or distribution evaluation ex vivo.

## Figures and Tables

**Table 1:** Regulatory opinions and acceptance of in vitro solubility and dissolution studies, permeability studies, cell culture models, and ex vivo models on the bioavailability of oral drugs.

Study Type	Regulatory Acceptance or Opinion
**Solubility**	▪ At least three pH values (pH 1.2, 4.5, and 6.8) should be evaluated. A drug substance is classified as highly soluble if the highest single therapeutic dose is completely soluble in aqueous media not more than 250 mL [72]. ▪ If the drug substance is not stable with >10% degradation during the solubility assessment, the drug substance cannot be classified for BCS Class [73]. ▪ Solubility is a vital factor for immediate-release (IR) products, especially when they are considered for biowaiver during drug approval [74].
**Dissolution**	▪ Drug dissolution studies are elementary to prove the immediate release of the drug when applying for a BCS-based biowaiver (BCS I and III only) [73]. ▪ Well-defined apparatus with agitation speeds [73]. ▪ The prolonged-release formulation should therefore be evaluated in vitro under various conditions, namely media, pH (normally pH range 1–7.5; if needed up to 8), and the use of biorelevant media is encouraged [75]. ▪ When drug release from MR formulations is investigated, in vitro studies of the release in alcohol solutions should be performed if the drug has higher solubility in ethanolic solutions than in water [76]. ▪ For authorization of MR products with several strengths (in the case of multi-particulate dosage forms/proportional tablets), if their release profiles are similar, the highest strength should be tested in vivo for food effect [76].
**Permeability on artificial membrane**	▪ Up to date, no guideline enlists or describes permeability studies for oral products involving artificial membranes.
**Cell culture models**	▪ Validation of Caco-2 permeability assay by markers with zero, low, moderate, and high permeability. Markers are enlisted in the guidelines. ▪ BCS classification of test drug is possible; a drug is considered BCS I or II when its permeability value is equal to or greater than that of the highly-permeable internal standard. ▪ The BCS classification by the Caco-2 cell line can be used only for drugs with passive transport [73].
**Ex vivo models**	▪ Currently, FDA guidelines enlist permeation studies using excised human or animal intestinal tissues [74].

**Table 2:** Regulatory opinions and acceptance of in vitro solubility and dissolution studies, permeability studies, cell culture models, and ex vivo models on the bioavailability of nasal/inhalation drugs.

Study Type	Regulatory Acceptance or Opinion
**Solubility**	▪ No guidelines are available about solubility studies. However, particle size, morphic form, and the state of solvation of the active substance can affect the bioavailability of a drug product as a result of different solubilities and/or rates of dissolution. Comparable data about particle size distribution, the morphic form of the particles, and the size and number of drug aggregates in the dosage form are recommended [105].
**Dissolution**	▪ Availability to the sites of action depends on the particle sizes and distribution patterns, as well as drug dissolution in the case of suspension products, absorption across mucosal barriers to nasal receptors, and rate of removal from the nose. The critical factors are drug release from the product and delivery to the mucosa [105]. ▪ For suspension products, drug particle size is important for the rate of dissolution and availability to sites of action within the nose. Therefore, drug particle size distribution and extent of aggregates should be characterized in formulation before actuation, and in the spray following actuation [105]. ▪ To assess the delivery profile of the product used in in vivo studies, the drug delivery rate and total drug delivered results should be provided for the batches used in these studies. A validated method (e.g., breath simulator), should be employed [106].
**Permeability on artificial membrane**	▪ No guidelines describe permeability studies for nasal or inhalation products.
**Cell culture models**	▪ No guidelines are available about cell culture models. The most frequently used cell lines for in vitro cell culture studies include RPMI2650, Calu-3, and 16HBE14o.
**Ex vivo models**	▪ No guidelines are available.

**Table 3:** Regulatory opinions and acceptance of in vitro dissolution studies, permeability studies, cell culture models, and ex vivo models on the bioavailability of transdermal drugs/products.

Study Type	Regulatory Acceptance or Opinion
Dissolution *	▪ In vitro drug release test is defined only for patches by regulator’s guidelines, the methods are described in Pharmacopeia (Ph. Eur., USP) [165,166]. ▪ Official pharmacopoeia methods of in vitro drug release testing: Paddle over Disk (Apparatus 5), Cylinder (Apparatus 6), or Reciprocating Holder (Apparatus 7) [165]. ▪ It is not correlated with in vivo, but it is necessary to be determined in the finished product release and shelf-life specification [166].
**Permeability on artificial membrane**	▪ In vitro release test (IVRT) is described as a permeability study for transdermal products involving artificial membranes. ▪ The artificial membrane used must have adequate properties to separate the product from the receptor medium and must not interfere with the flow of the active substance or bind that. ▪ In the case of receptor medium, sink conditions should be confirmed. The maximum concentration of the API in the receptor medium achieved during the experiment does not exceed 30% of its maximum solubility in the receptor medium [167].
**Cell culture models**	▪ Up to date, no guidelines enlist or describe permeability studies involving cell culture for transdermal products.
**Ex vivo models**	▪ Currently, FDA and EMA guidelines enlist permeation studies (IVPT) using excised human or animal skin tissues. ▪ The most accepted is adult human skin, which does not contain tattoos, any diseases, and a hairy surface. ▪ The use of aqueous buffers as a receptor/release medium is recommended, which does not damage the integrity of the tissue, otherwise, it is necessary to check it after the test. ▪ During the test, 12 parallel experiments with samples taken from the same place from different donors are required to prove reproducibility, the test must be performed at 32 °C for 24 h. ▪ Only tape stripping is accepted by the guidelines for testing the accumulation of the active substance in the tissue, RAMAN, and microdialysis methods can only provide additional information [167].

* To use for patches.

**Table 4:** Regulatory opinions and acceptance of in vitro dissolution studies, permeability studies, cell culture models, and ex vivo models on the bioavailability of ophthalmic drugs/products.

Study Type	Regulatory Acceptance or Opinion
**Dissolution**	▪ In vitro release studies can be performed in case of qualitative and quantitative sameness of the products. ▪ The methodology used for in vitro drug release testing should be able to discriminate the effect of process and variability in the production of the test formulation [171,172]. ▪ No concrete method is described; generally, Franz diffusion cell or dialysis tests are performed.
**Permeability on artificial membrane**	▪ To date, no guidelines enlist or describe permeability studies involving artificial membranes for ophthalmic products.
**Cell culture models**	▪ To date, no guidelines enlist or describe permeability studies involving cell culture for ophthalmic products.
**Ex vivo models**	▪ To date, no guidelines enlist or describe permeability studies involving ex vivo models for ophthalmic products.

## Data Availability

Not applicable.
